# Correction: The epidemiology of Mayaro virus in the Americas: A systematic review and key parameter estimates for outbreak modelling

**DOI:** 10.1371/journal.pntd.0011034

**Published:** 2023-01-04

**Authors:** Edgar-Yaset Caicedo, Kelly Charniga, Amanecer Rueda, Ilaria Dorigatti, Yardany Mendez, Arran Hamlet, Jean-Paul Carrera, Zulma M. Cucunubá

There is an error in Table 3. The mean for the intrinsic incubation period should be 3.0 (95% CrI: 2.4–4.1) days not 3.0 (95% CrI: 2.2–3.8) days. The standard deviation for the intrinsic incubation period should be 0.3 (95% CrI: 0.0–2.1) days not 1.2 (95% CrI: 1.0–1.7) days. Please see the correct Table 3 below.

**Table 3:** Estimates of the mean and standard deviation of the generation time distribution and its components.

	Mean (95% CrI)	Standard deviation (95% CrI)	Source
**Intrinsic incubation period (days)**	3.0 (2.4–4.1)	0.3 (0.0–2.1)	estimated
**Time to viral clearance (days)**	3.9 (3.5–4.4)	1.0 (0.8–1.2)	estimated
**Human-to-mosquito generation time (days)**	3.4 (2.0–4.8)	0.7 (0.5–1.1)	estimated
**Extrinsic incubation period (days)**	9.4 (8.4–10.7)	4.6 (3.3–6.7)	estimated
**Mosquito life time (days)** **(as for *Aedes aegypti*)**	5.3 (fixed)	1.4 (fixed)	[32]
**Mosquito-to-human generation time (days)**	11.9 (8.6–16.3)	6.2 (4.2–9.5)	estimated
**Mayaro virus generation time (days)**	15.2 (11.7–19.8)	6.2 (4.2–9.5)	estimated

There is an error on page 6 of S1 Text. The text says, “We estimated a mean incubation period μ_IP_ of 3.0 (95% CrI: 2.2–3.8) days and a standard deviation σ_IP_ of 1.2 (95% CrI: 1.0–1.7) days.” It should say, “We estimated a mean incubation period μ_IP_ of 3.0 (95% CrI: 2.4–4.1) days and a standard deviation σ_IP_ of 0.3 (95% CrI: 0.0–2.1) days.” Please view the correct S1 Text below.

## Supporting information

S1 Text Fig A. Flowchart showing the selection of studies. **Fig B. Viral load detected in plasma in Mayaro infected cases per day post symptoms onset.** Mean and range for 21 patients are shown. **Fig C. Maximum likelihood EIP probability density function (left) and cumulative distribution function (right).** The aggregated proportion of mosquitoes that tested positive at the relative days post-infection are shown as bars. **Table A. Boolean algorithms for literature search. Table B. Data classification of MAYV studies in humans. Table C. Values used in estimate_R() function in EpiEstim package. Table D. Characteristics of Mayaro fever case reports. Table E. Characteristics of Mayaro fever cases included in the intrinsic incubation period analysis (N = 15). Table F. Characteristics of hospital-based surveillance studies included in the analysis. Table G. Characteristics of MAYV cross-sectional seroprevalence studies. Table H. Studies with possible evidence of MAYV transmission.** These studies were not classified in other categories but strongly indicate presence of MAYV. **Table I. Studies that detected MAYV in animals. Table J. Full genomes of MAYV included in the phylogenetic analysis. Table K. Nucleotide substitution models.** The best-fitting model is in bold.
(DOCX)
