# Supplementary material for: The epidemiology of Mayaro virus in the Americas: A systematic review and key parameter estimates for outbreak modelling
Source: PLoS Negl Trop Dis. 2021 Jun 3;15(6):e0009418. doi: 10.1371/journal.pntd.0009418 (PMC8205173; doi:10.1371/journal.pntd.0009418)
Supplement: S1 Text — Fig A. Flowchart showing the selection of studies. Fig B. Viral load detected in plasma in Mayaro infected cases per day post symptoms onset. Mean and range for 21 patients are shown. Fig C. Maximum likelihood EIP probability density function (left) and cumulative distribution function (right). The aggregated proportion of mosquitoes that tested positive at the relative days post-infection are shown as bars. Table A. Boolean algorithms for literature search. Table B. Data classification of MAYV studies in humans. Table C. Values used in estimate_R() function in EpiEstim package. Table D. Characteristics of Mayaro fever case reports. Table E. Characteristics of Mayaro fever cases included in the intrinsic incubation period analysis (N = 15). Table F. Characteristics of hospital-based surveillance studies included in the analysis. Table G. Characteristics of MAYV cross-sectional seroprevalence studies. Table H. Studies with possible evidence of MAYV transmission. These studies were not classified in other categories but strongly indicate presence of MAYV. Table I. Studies that detected MAYV in animals. Table J. Full genomes of MAYV included in the phylogenetic analysis. Table K. Nucleotide substitution models. The best-fitting model is in bold. (DOCX) [file pntd.0009418.s002.docx]

**S1 Text**

**The epidemiology of Mayaro virus in the Americas:**

**A systematic review and key parameter estimates for outbreak modelling**

Edgar-Yaset Caicedo^1+^, Kelly Charniga^2+^, Amanecer Rueda^1^, Ilaria Dorigatti^2^, Yardany Mendez^1^, Arran Hamlet^2^, Jean-Paul Carrera^3,4^, Zulma M. Cucunubá^2*^

^1^Universidad Pedagógica y Tecnológica de Colombia, Tunja, Colombia

^2^MRC Centre for Global Infectious Disease Analysis (MRC-GIDA), Imperial College London, London, UK

^3^Department of Zoology, University of Oxford, Oxford, UK

^4^Department of Research in Virology and Biotechnology, Gorgas Memorial Institute of Health Studies, Panama City, Panamá

+ These authors contributed equally to this work

* [zulma.cucunuba@imperial.ac.uk](mailto:zulma.cucunuba@imperial.ac.uk)

**Supplementary methods for systematic review**

Records identified by searching databases (n = 1222)

Records after duplicates removed

(n = 713)

Records screened

(n = 713)

Unrelated records excluded

(n = 573)

Full-text articles assessed for eligibility

(n = 140)

Full text excluded because inclusion criteria not met (n = 50)

Studies included in analysis

(n = 76)

Identification

Screening

Eligibility

Included

**Fig A. Flowchart showing the selection of studies.**

**Table A. Boolean algorithms for literature search.**

| Database | Algorithm* | Number of titles | Dates |
| --- | --- | --- | --- |
| Web of knowledge | ((mayaro virus OR MAYV OR uruma)) | 168 | 11 Jan 2019 |
| PubMed | ((mayaro[All Fields] AND ("viruses"[MeSH Terms] OR "viruses"[All Fields] OR "virus"[All Fields])) OR uruma[All Fields]) OR MAYV[All Fields] | 434 | 11 Jan 2019 |
| LILACS | (tw:(mayaro virus OR fiebre de mayaro OR MAYV OR uruma)) | 248 | 11 Jan 2019 |
| EMBASE | mayaro virus.af  MAYV.af  uruma.ab  #1 OR #2 OR #3 | 213 | 15 Jan 2019 |
| Google  Scholar | (Mayaro Virus OR MAYV) | 159 | 11 Jan 2019 |
| Total |  | 1222 |  |

*No restrictions were used on any of the search terms.

**Table B. Data classification of MAYV studies in humans.**

| Study type | Definition |
| --- | --- |
| Case reports | A detailed report describing one or more confirmed cases.  Tests: polymerase chain reaction (PCR), IgM, or isolates or a combination of these with symptom surveillance, or seroconversion by IgG (with evidence of previous IgG negative), or an increased titre > 4 times the previous sample. |
| Outbreaks | An outbreak of confirmed and suspected cases is reported in detail (location, period of time, etc).  Tests: PCR, IgM, or isolates or a combination of these with symptom surveillance or seroconversion by IgG (with evidence of previous IgG negative) or an increased titre > 4 times to previous sample, with or without reported symptoms. |
| Hospital-based surveillance | A study that is performed in a health facility with symptomatic patients.  Tests: PCR, IgM (HI, ELISA), or isolates or a combination of these with symptom surveillance or seroconversion by IgG (HI, ELISA, complement fixation), (with evidence of previous IgG negative) or an increased titre of > 4 times the previous sample. IgG by ELISA or HI only if the samples tested negative against all other alphaviruses’ antigens. The health facilities were classified according to their location into urban, rural, and unknown location. |
| Cross-sectional seroprevalence | A study conducted in a community of asymptomatic people that is representative of the general population of that community.  Tests: antibodies against MAYV using one or more of the following techniques: platelet reaction neutralisation test (PRNT), neutralisation test (NT), ELISA or hemagglutination inhibition (HI). |
|  |  |
| Others | Not classified in other categories but strongly indicates MAYV presence. |

**Supplementary methods for generation time and time-varying reproduction number**

**Natural history parameter estimates**

**Viral load data.** We found one paper that reported the absolute quantification of Mayaro RNA viral copies in plasma following the onset of symptoms [1]. This paper reported the duration of viremia in 21 patients from whom Mayaro virus (MAYV) was isolated. Viremia data was reported on the log_10_ scale and presented as mean values and ranges (Fig B).

Mayaro viral loads in humans are comparable to those of Zika [2].

**Fig B. Viral load detected in plasma in Mayaro infected cases per day post symptoms onset.** Mean and range for 21 patients are shown. Adapted from [1].

**Human to mosquito generation time.** The human-to-mosquito generation time is the time between human infection and a mosquito taking an infectious blood meal. It is composed of the intrinsic incubation period (the time between human infection and symptoms onset) and the time from symptoms onset to viral clearance.

**Intrinsic incubation period.** We found 10 peer-reviewed articles that reported information on time of exposure to MAYV and time of symptoms onset in humans. From these, we extracted data on 15 cases of Mayaro fever. All were infected while traveling to endemic areas. Most cases were infected after 2004, no cases were reported in children, and of the 11 studies that reported the sex of patients, six were males and five were females.

We excluded articles if they did not report quantitative information on time of exposure and symptoms onset. We used the exact timing of exposure whenever possible. When this information was not reported, we used the information provided to bound the time of exposure. Following Lessler et al., we bounded the time of MAYV infection by the earliest and latest potential times of exposure [3].

A doubly interval censored dataset was constructed for the incubation period and the distribution was fitted using the methods described in [3,4]. Following Lessler et al. [3], we assumed that the incubation period of MAYV followed a log-normal distribution and used the Metropolis-Hastings Markov chain Monte Carlo (MCMC) algorithm for calibration.

We estimated a mean incubation period μ_IP_ of 3.0 (95% CrI: 2.2-3.8) days and a standard deviation σ_IP_ of 1.2 (95% CrI: 1.0-1.7) days.

**Time to viral clearance.** Following [2], we assumed the time to viral clearance is Gamma distributed with shape parameter $\alpha_{C}$ and scale parameter $\beta_{C}$. We estimated $\alpha_{C}$ and $\beta_{C}$ using the Metropolis-Hastings MCMC algorithm. In Table 3 of the main text, we report the mean ($\mu_{C}=\alpha_{C}\beta_{C}$) and standard deviation ($\sigma_{C}=\alpha_{C}\beta_{C}^{2}$) of the time to viral clearance.

We also assumed that infectiousness in Mayaro infection is similar to Zika infection, beginning 1.5 days before symptoms onset and ending 1.5-2 days before viral levels can no longer be detected. To obtain the human generation time from the intrinsic incubation period and time to viral clearance, we linearly scaled the time dependence of the distribution by a factor $s=\left( \mu_{IP}-1.5 \right)/\mu_{IP}$. This results in a human generation time with mean $\mu_{h}=s\left( \mu_{IP}+\mu_{C} \right)$ and standard deviation $\sigma_{h}=s\sqrt{\sigma_{IP}^{2}+\sigma_{C}^{2}}$.

**Extrinsic incubation period.** We found five peer-reviewed articles that reported the susceptibility of mosquitoes to MAYV [5-9]. Only three articles [6,8,9] reported enough information on the number of mosquitoes tested on each day post-infection, which is necessary to estimate the extrinsic incubation period. Across these studies, seven different species of mosquitoes and three different strains of MAYV were used. Due to limited data, we combined information across all species of mosquitoes and viral strains. We dropped *Cx. quinquefasciatus* from the pooled analysis because it had null transmission rates. We also dropped observations on day 14 from [6] because the observed frequency did not match with the pattern observed in the rest of the data. We defined the extrinsic incubation period as the time between infection and MAYV reaching the salivary glands.

As in [2], we assumed that the extrinsic incubation period is Gamma distributed with shape parameter k_EIP_ and scale parameter θ_EIP_. Using a Binomial likelihood function to estimate the probability that a mosquito is infectious by day *t*, we obtain mean posterior estimates of k_EIP_ = 4.5 (95% CrI: 2.4-7.3) and θ_EIP_ = 2.3 (95% CrI: 1.2-4.3). This results in a mean EIP of 9.4 (95% CrI: 8.4-10.7) days with a standard deviation of 4.6 (95% CrI: 3.3-6.7) days (main text Table 3). Fig C shows the fitted probability density function, cumulative distribution function, and observed data.


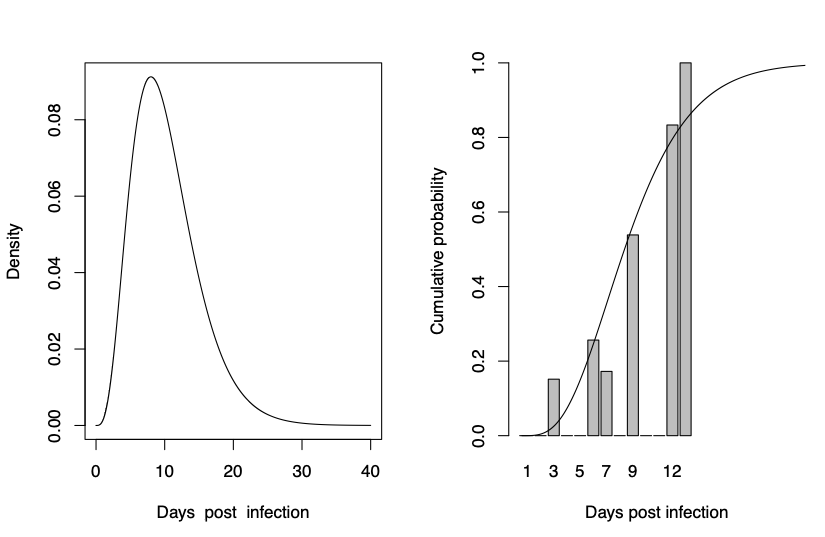


**Fig C.** **Maximum likelihood EIP probability density function (left) and cumulative distribution function (right).** The aggregated proportion of mosquitoes that tested positive at the relative days post-infection are shown as bars.

**Mosquito-to-human generation time.** We use the same methods as [2] to estimate the mosquito-to-human generation time (the time between a mosquito being infected and it infecting a human) from the estimated EIP and the mosquito daily mortality rate. As in [2], we assume that the mosquito mortality rate $ɛ$ is Gamma distributed with a mean of 0.2/day and a standard deviation of 0.05/day.

We define the probability density function of the mosquito-to-human generation time as

$$h\left( t \right)= \frac{f^{'}\left( t \right)e^{-ɛt}}{\int_{0}^{\infty} f^{'}\left( t^{'} \right)e^{-ɛt^{'}}dt'}$$

where $f'\left( t \right)$represents the density function of the EIP (the probability density of a mosquito being infectious at time $t$). We estimate the mean 𝜇_m_ and standard deviation σ_m_ of the mosquito-to-human generation time numerically, i.e. sampling the shape parameter k_EIP_ and scale parameter θ_EIP_ from their posterior distributions and $ɛ$ from the Gamma distribution with mean of 0.2/day and standard deviation of 0.05/day.

We estimated a mean mosquito-to-human generation time of 11.9 (95% CrI: 8.6 - 16.3) days and a standard deviation of 6.2 (95% CrI: 4.2 - 9.5) days (main text Table 3).

**Generation time of MAYV.** Combining the estimates of the human-to-mosquito generation time with those of the mosquito-to-human generation time, we estimate that the distribution of the generation time of MAYV (i.e. the time between infection of a human case and infection of the secondary human cases that case causes) has a mean of 15.2 (95% CrI: 11.7 - 19.8) days and a standard deviation of 6.2 (95% CrI: 4.2 - 9.5) days (main text Table 3).

**Estimates of the reproduction number, R**

We estimated the instantaneous reproduction number *R* for the 1954-1955 MAYV outbreak in Santa Cruz, Bolivia using the weekly case incidence and the generation time distribution estimated in the previous section.

The instantaneous reproduction number *R* was calculated over 4-week sliding windows using the EpiEstim package in R software [10]. The instantaneous reproduction number *R* of each time window is calculated as the median of the weekly instantaneous reproduction number weighted by the weekly incidence. *R* values were plotted in the middle of the 4-week time window used to calculate each estimate. We did not include the seven cases reported at the end of the outbreak in our estimation of *R*. Based on our estimate of the generation time distribution, we do not believe these cases are part of the same transmission chain as the earlier cases.

The analysis was performed in R (version 3.5.3). The *‘uncertain_si’* option was selected in the estimate_R() function of EpiEstim, using the mean and standard deviation of the generation time from Table 3 in the main text and a prior distribution for R with a mean and standard deviation of 5. All values can be found in Table C. The uncertain_si method takes into account uncertainty on the serial interval distribution as described in Cori et al. [11]. Briefly, the mean μ and standard deviation σ of the serial interval are permitted to vary according to truncated normal distributions. The values in Table C were used to sample n1 pairs of mean and standard deviations from their respective truncated normal distributions. For each pair, a sample of size n2 was drawn from the posterior distribution of the reproduction number over each time window. n2 was drawn conditionally on the serial interval distribution obtained. A sample of size n1xn2 of the joint posterior distribution of the reproductive number over each time window was generated after pooling.

**Table C. Values used in estimate_R() function in EpiEstim package.**

| Parameter | Days* |
| --- | --- |
| mean_si | 15.2 |
| std_mean_si | 2.1 |
| min_mean_si | 7.0 |
| max_mean_si | 29.5 |
| std_si | 6.3 |
| std_std_si | 1.3 |
| min_std_si | 3.0 |
| max_std_si | 17.0 |

*All values were divided by 7 for weekly data.

**Supplementary results for systematic review**

**Table D. Characteristics of MAYV case reports.**

| Ref | Year of detection | Country | State | Town/City | Diagnostic method* | Diagnostic certainty | Cases | Zone | Origin |
| --- | --- | --- | --- | --- | --- | --- | --- | --- | --- |
| [12] | 2012 | Bolivia | Beni | Rurrenabaque | IgM/IgG NT and IA | confirmed | 1 | rural | foreign |
| [13] | 2000 | Brazil | Mato Grosso | Camapua | Culture, RT-PCR | confirmed | 3 | rural | foreign |
| [14] | 2004 | Brazil | Acre | Acrelandia | RT-PCR | confirmed | 1 | rural | native |
| [15] | 2009 | Brazil | Amazonas | Barcelos | IgM ELISA | potential | 1 | rural | foreign |
| [16] | 2013 | Brazil | Para |  | IgM | potential | 2 | rural | native |
| [17] | 2015 | Brazil | Sao Paulo | Sao Jose do Rio Preto | RT-PCR | confirmed | 1 | rural | foreign |
| [18] | 2015 | Brazil | Para | Portal | RT-PCR | confirmed | 1 | urban | native |
| [19] | 1996 | French Guiana |  |  | Culture, RT-PCR | confirmed | 1 | rural | native |
| [20] | 1998 | French Guiana | Cayenne | Cayenne | IgM ELISA | potential | 1 | urban | native |
| [21] | 2012 | French Guiana | Cayenne | Kourou | IgM ELISA | potential | 3 | mixed | native |
| [22] | 2013 | French Guiana |  |  | RT-PCR | potential | 1 | rural | foreign |
| [23] | 2015 | French Guiana | Cayenne | Roura | RT-PCR | confirmed | 1 | rural | foreign |
| [24] | 2014 | Haiti | Oest | Port-au-Prince | RT-PCR | confirmed | 1 | urban | native |
| [25] | 2001 | Mexico | Tamaulipas | Tampico | IgM ELISA | potential | 1 | urban | native |
| [25] | 2001 | Mexico | Veracruz | Coatzacoalco | IgM ELISA | potential | 1 | urban | native |
| [26] | 1995 | Peru | Loreto | Iquitos | Culture, IgM ELISA, PCR | confirmed | 2 | urban | foreign |
| [26] | 1995 | Peru | Loreto | Iquitos | Culture, IgM ELISA, PCR | confirmed | 13 | mixed | native |
| [26] | 1995 | Peru | Loreto | Yurimaguas | Culture, IgM ELISA, PCR | confirmed | 1 | mixed | native |
| [26] | 1995 | Peru | San Martin | Tocache | Culture, IgM ELISA, PCR | confirmed | 1 | mixed | native |
| [26] | 1995 | Peru | Ucayali | Pucallpa | Culture, IgM ELISA, PCR | confirmed | 6 | mixed | native |
| [26] | 1995 | Peru | Huanuco | Huanuco | Culture, IgM ELISA, PCR | confirmed | 3 | mixed | native |
| [26] | 1995 | Peru | Cusco | Quillabamba | Culture, IgM ELISA, PCR | confirmed | 1 | mixed | native |
| [26] | 1995 | Peru | Tumbes | Tumbes | Culture, IgM ELISA, PCR | confirmed | 2 | mixed | native |
| [27] | 2011 | Peru | San Martin | Tarapoto | IgM/IgG NT and IA | confirmed | 1 | rural | foreign |
| [28] | 2008 | Surinam |  |  | IgM ELISA | potential | 2 | rural | foreign |
| [29] | 1954 | Trinidad and Tobago | Mayaro | Cats Hill | Culture | confirmed | 5 | rural | native |
| [30] | 2000 | Venezuela | Miranda | Padron Agriculture | IgM ELISA | potential | 4 | rural | native |

NT: neutralization test, IA: immunofluorescence assay.

**Table E. Characteristics of Mayaro fever cases included in the intrinsic incubation period analysis (N = 15).**

| Ref | Age | Sex | Place of origin | Probable location infected | Year exposed | Exposure window (days) | Days to symptoms onset (min-max) |
| --- | --- | --- | --- | --- | --- | --- | --- |
| [23] | 30 | Male | France | French Guiana | - | 6 | 1-8 |
| [22] | 44 | Female | Germany | French Guiana | 2013 | 18 | 1-20 |
| [16] | 52 | Female | Netherlands | Brazil | 2013 | 26 | 0-26 |
| [12] | 20 | Female | Germany | Bolivia | 2012 | 10 | 1-12 |
| [27] | 27 | Male | Switzerland | Peru | 2011 | 14 | 0-14 |
| [15] | Late 20s | Male | France | Brazil | 2010 | 14 | 0-14 |
| [13] | 36  59  74 | Male  Male  Male | SP, Brazil  SP, Brazil  SP, Brazil | MS, Brazil  MS, Brazil  MS, Brazil | 2000  2000  2000 | 8  9  8 | 0-8  0-10  0-8 |
| [30] | 26-58  26-58  26-58 | -  -  - | -  -  - | Venezuela  Venezuela  Venezuela | 2000  2000  2000 | 1  1  1 | 2-4  2-4  2-4 |
| [26] | 48  29 | Female  Female | USA  USA | Peru  Peru | 1996  1997 | 14  75 | 0-15  0-75 |
| [1] | - | - | Brazil | PA, Brazil | 1978 | 7 | 0-7 |

**Table F. Characteristics of hospital-based surveillance studies included in the analysis.**

| Ref | Study years | Country | State | Town/city | Diagnostic method* | Other arboviruses studied | Total | No. positive | % positive | Zone | Origin |
| --- | --- | --- | --- | --- | --- | --- | --- | --- | --- | --- | --- |
| [31] | 2000-2007 | Bolivia | Santa Cruz | Santa Cruz | Culture, RT PCR, IgM ELISA | VEEV, MURV, CARV, EEV, YFV, OROV, GUAV, DENV | 1280 | 10 | 0.8% | unknown | native |
| [31] | 2004-2006 | Bolivia | Beni | Magdalena | Culture, RT PCR, IgM ELISA | VEEV, MURV, CARV, EEV, YFV, OROV, GUAV, DENV | 173 | 1 | 0.6% | unknown | native |
| [31] | 2004-2007 | Bolivia | Santa Cruz | Concepcion | Culture, RT PCR, IgM ELISA | VEEV, MURV, CARV, EEV, YFV, OROV, GUAV, DENV | 380 | 3 | 0.8% | unknown | native |
| [31] | 2005-2007 | Bolivia | Cochabamba | Cochabamba | Culture, RT PCR, IgM ELISA | VEEV, MURV, CARV, EEV, YFV, OROV, GUAV, DENV | 256 | 10 | 3.9% | unknown | native |
| [32] | 1998-1999 | Brazil | Amazonas | Manaus | IgM ELISA | DENV, OROV | 8577 | 8 | 0.1% | urban | native |
| [33] | 2007-2008 | Brazil | Amazonas | Manaus | Culture, RT PCR, IgM ELISA | no | 631 | 33 | 5.2% | urban | native |
| [34] | 2009 | Brazil | Para | Novo Progresso | IgM ELISA and HI | DENV, YFV, OROV | 744 | 28 | 3.8% | unknown | native |
| [34] | 2009 | Brazil | Para | Trairão | IgM ELISA and HI | DENV, YFV, OROV | 654 | 49 | 7.5% | unknown | native |
| [35] | 2011-2013 | Brazil | Goias | Goiania | IgM EIA-ICC ELISA | DENV, OROV | 647 | 6 | 0.9% | unknown | native |
| [36] | 2011-2012 | Brazil | Mato Grosso | Sinop | RT-PCR | UNAV, SFV, Getah virus, Ross River virus | 200 | 6 | 3.0% | urban | native |
| [37] | 2011-2012 | Brazil | Mato Grosso |  | RT-PCR | AURV, WEEV, EEEV, DENV | 604 | 15 | 2.5% | unknown | native |
| [38] | 2014-2015 | Brazil | Goias |  | IgM MAC-ELISA and HI | DENV, CHIKV | 75 | 15 | 20.0% | unknown | foreign |
| [39] | 2015-2016 | Brazil | Mato Grosso |  | RT-PCR | DENV, YF, SLEV, ILHV, ROCV, WNV, EEEV, WEEV, VEEV, CHIKV | 453 | 34 | 7.5% | unknown | native |
| [40] | 2016-2017 | Brazil | Piaui | Parnaiba | RT-PCR | DENV, CHIKV | 578 | 1 | 0.2% | urban | native |
| [31] | 2003-2007 | Ecuador | Guayas | Guayaquil | Culture, RT PCR, IgM ELISA | VEEV, MURV, CARV, EEV, YFV, OROV, GUAV, DENV | 350 | 1 | 0.3% | unknown | native |
| [41] | 2003-2016 | French Guiana | Cayenne |  | RT-PCR or IgM ELISA | no | 412 | 9 | 2.2% | urban | native |
| [31] | 2000-2007 | Peru | Cusco | Cusco | Culture, RT PCR, IgM ELISA | VEEV, MURV, CARV, EEV, YFV, OROV, GUAV, DENV | 826 | 4 | 0.5% | unknown | native |
| [42] | 2000-2001 | Peru | Morropon | Salitral | IgM-ELISA | DENV, YFV, OROV, VEEV | 65 | 1 | 1.5% | unknown | native |
| [31] | 2004-2007 | Peru | Loreto | Yurimaguas | Culture, RT PCR, IgM ELISA | VEEV, MURV, CARV, EEV, YFV, OROV, GUAV, DENV | 1452 | 11 | 0.8% | unknown | native |
| [31] | 2004-2007 | Peru | Loreto | Iquitos | Culture, RT PCR, IgM ELISA | VEEV, MURV, CARV, EEV, YFV, OROV, GUAV, DENV | 10739 | 48 | 0.5% | unknown | native |
| [31] | 2004-2007 | Peru | Madre de Dios | Puerto Maldonado | Culture, RT PCR, IgM ELISA | VEEV, MURV, CARV, EEV, YFV, OROV, GUAV, DENV | 1215 | 10 | 0.8% | unknown | native |
| [43] | 2010-2013 | Peru | Loreto | Iquitos | Culture, RT-PCR, seroconversion of IgM | VEEV, OROV, GUAV, DENV | 2094 | 16 | 0.8% | unknown | native |

*****HI: hemagglutination inhibition, NT: neutralization test, IA: immunofluorescence assay.

**AURV: Aura virus, CARV: Caraparu virus, CHIKV: chikungunya virus, DENV: dengue virus, EEEV: Eastern equine encephalitis virus, GUAV: Guaroa virus, ILHV: Ilheus virus, MADV: Madariaga virus, MUCV: Mucambo virus, OROV: Oropuche virus, ROCV: Rocio virus, SLEV: Saint Louis encephalitis virus, UNAV: Una virus, VEEV: Venezuelan equine encephalitis virus, WEEV: Western equine encephalitis virus, YFV: yellow fever virus, ZIKV: Zika virus.

**Table G. Characteristics of MAYV cross-sectional seroprevalence studies in humans.**

| Ref | Study year | Country | State | Diagnostic method* | Total | No. positive | Sero-prevalence | Age stratified | Zone | Indigenous community | Population-based |
| --- | --- | --- | --- | --- | --- | --- | --- | --- | --- | --- | --- |
| [44] | 1997 | Ecuador | Morona-Santiago | ELISA | 91 | 42 | 46.2% | yes | rural | yes, in military service | no |
| [45] | 2007 | Brazil | Amazonia | ELISA | 270 | 119 | 44.1% | yes | rural | no | yes |
| [46] | 1965 | Brazil | Para | HI | 221 | not reported | 37.0% | yes | rural | yes, Gorotire | yes |
| [46] | 1966 | Brazil | Para | HI | 178 | not reported | 47.0% | yes | rural | yes, Tiriyo | yes |
| [46] | 1969 | Brazil | Para | HI | 189 | not reported | 49.0% | yes | rural | yes, Mekranoti | yes |
| [46] | 1970 | Brazil | Para | HI | 69 | not reported | 20.0% | yes | rural | yes, Kuben KK | yes |
| [46] | 1970 | Brazil | Para | HI | 102 | 47 | 46.1% | yes | rural | yes, Xikrin | yes |
| [19] | 1996 | French Guiana |  | HI | 1962 | 124 | 6.3% | yes | rural | no | yes |
| [47] | 1957 | Guyana | Rupununi Savannah | NT | 221 | 126 | 57.0% | yes | rural | yes, Amerindians of the Rupununi | no |
| [47] | 1957 | Trinidad and Tobago |  | NT | 615 | 69 | 11.2% | yes | rural | no | yes |
| [48] | 1960 | Colombia | Santander | NT | 176 | 38 | 21.6% | yes | rural | no | no |
| [49] | 2011 | Peru | Iquitos | PRNT and IgG ELISA | 70 | 38 | 54.3% | yes | rural | yes, Nueva Esperanza | no |
| [50] | 1966 | Colombia | Amazonas | HI and NT | 396 | 76 | 19.2% | yes | rural | some | no |
| [51] | 1956 | Guyana | Rupununi Savannahs | HI and NT | 176 | 38 | 21.6% | yes | rural | yes, Macusi and wapisiani tribes | no |
| [52] | 1964 | Surinam | Brokopondo | NT | 132 | 88 | 66.7% | yes | rural | no | yes |
| [53] | 1960 | Surinam |  | HI and NT | 500 | 8 | 1.6% | no | rural | no | no |
| [54] | 1965 | Peru |  | HI and NT | 100 | 68 | 68.0% | no | rural | no | no |

*****HI: hemagglutination inhibition, NT: neutralization test, PRNT: plaque reduction neutralization test, ELISA: enzyme-linked immunosorbent assay.

**Table H. Studies with possible evidence of MAYV transmission in humans.**

| Ref | Study years | Country | State | Town/city | Source population | Showed symptoms? | Diagnostic method* | Other arboviruses studied** | Total | No. positive | % positive | Zone | Origin |
| --- | --- | --- | --- | --- | --- | --- | --- | --- | --- | --- | --- | --- | --- |
| [55] | 1999 | Bolivia |  |  | hospital | yes | serology | no | 1 | 1 | 100% | rural | foreign |
| [56] | 1955 | Brazil | Para | Abaetetuba | community | some | NT to SLF | no | 36 | 5 | 13.9% | rural | native |
| [56] | 1955 | Brazil | Para | Altamira | community | some | NT to SLF | no | 24 | 1 | 4.2% | rural | native |
| [56] | 1955 | Brazil | Para | Belterra | community | some | NT to SLF | no | 17 | 5 | 29.4% | rural | native |
| [56] | 1955 | Brazil | Para | Cametá | community | some | NT to SLF | no | 29 | 7 | 24.1% | rural | native |
| [56] | 1955 | Brazil | Para | Capim River | community | some | NT to SLF | no | 33 | 1 | 3.0% | rural | native |
| [56] | 1955 | Brazil | Para | Fordlandia | community | some | NT to SLF | no | 11 | 1 | 9.1% | rural | native |
| [56] | 1955 | Brazil | Amazonas | Labrea | community | some | NT to SLF | no | 24 | 7 | 29.2% | rural | native |
| [56] | 1955 | Brazil | Amazonas | Manaus | community | some | NT to SLF | no | 15 | 1 | 6.7% | rural | native |
| [56] | 1955 | Brazil | Para | Obidos | community | some | NT to SLF | no | 14 | 2 | 14.3% | rural | native |
| [56] | 1955 | Brazil | Para | Belem | community | some | NT to SLF | no | 136 | 3 | 2.2% | rural | native |
| [46] | 1965 | Brazil | Para | Gorotire | community | no | HI | EEEV, MUCV, PIXV, UNAV, AURV, WEEV, YFV, ILHV, BUSV, SLEV, BUNV, MAGV, GUAV | 221 | 90 | 40.7% | rural | native |
| [46] | 1966-1970 | Brazil | Para | Tiriyo | community | no | HI | EEEV, MUCV, PIXV, UNAV, AURV, WEEV, YFV, ILHV, BUSV, SLEV, BUNV, MAGV, GUAV | 217 | 127 | 58.5% | rural | native |
| [57] | 1967 | Brazil | Mato Grosso | Simao Lopes | community | no | HI | ILHV, YFV, BUNV | 155 | 31 | 20.0% | rural | native |
| [57] | 1967 | Brazil | Mato Grosso | Sao Marcos | community | no | HI | ILHV, YFV, BUNV | 257 | 45 | 17.5% | rural | native |
| [46] | 1969-1972 | Brazil | Para | Mekranoti | community | no | HI | EEEV, MUCV, PIXV, UNAV, AURV, WEEV, YFV, ILHV, BUSV, SLEV, BUNV, MAGV, GUAV | 190 | 93 | 48.9% | rural | native |
| [46] | 1970 | Brazil | Para | Kuben Kran Kegn | community | no | HI | EEEV, MUCV, PIXV, UNAV, AURV, WEEV, YFV, ILHV, BUSV, SLEV, BUNV, MAGV, GUAV | 69 | 16 | 23.2% | rural | native |
| [46] | 1970-1972 | Brazil | Para | Krikin | community | no | HI | EEEV, MUCV, PIXV, UNAV, AURV, WEEV, YFV, ILHV, BUSV, SLEV, BUNV, MAGV, GUAV | 102 | 47 | 46.1% | rural | native |
| [58] | 1972 | Brazil | Para | Altamaria | community | yes | HI seroconversion | SELV, WEEV, OROV, GUAV | 832 | 12 | 1.4% | rural | native |
| [59] | 1984 | Brazil | Bahia | Corte de Pedra | hospital | no | HI | WEEV, EEV, MUCV, YFV, BUSV, ILHV, SLEV, Cacipacore virus, ROCV, DENV, Itaporanga virus, Tacaiuma virus, Iaco virus, GUAV, OROV, Utinga virus | 288 | 1 | 0.3% | rural | foreign |
| [60] | 1999-2000 | Brazil | Acre | Rio Branco | community | no | HI seroconversion | MUCV, ILHV, ROCV, DENV, SLEV, YFV, OROV, CARV, Catu virus | 178 | 12 | 6.7% | mixed | native |
| [61] | 2007-2008 | Brazil | Para | Juruti | community | yes | HI | no | 1597 | 20 | 1.3% | rural | native |
| [45] | 2007 | Brazil | Amazonia |  | community | no | IgG ELISA | no | 270 | 119 | 44.1% | rural | native |
| [61] | 2007-2008 | Brazil | Para | Juruti | community | yes | IgM MAC-ELISA | DENV, YFV, OROV | 102 | 5 | 4.9% | rural | native |
| [44] | 1997 | Ecuador | Morona-Santiago |  | community | no | IgG ELISA | no | 91 | 42 | 46.2% | rural | military |
| [19] | 1996 | French Guiana |  |  | community | no | HI | CHIKV | 1305 | 115 | 8.8% | mixed | native |
| [62] | 2010 | Panama | Darien |  | community | some | IgM-ELISA | MADV, VEEV | 72 | 1 | 1.4% | rural | native |
| [63] | 1965 | Peru | Ucayalli | Pucallpa | community | no | HI | AURV, EEEV, MUCV, PIXU, UNAV, VEEV, WEEV | 546 | 164 | 30.0% | mixed | native |
| [63] | 1965 | Peru | Huanuco | TingoMaria | community | no | HI | AURV, EEEV, MUCV, PIXU, UNAV, VEEV, WEEV | 517 | 202 | 39.1% | mixed | native |
| [64] | 2006-2008 | Peru | Loreto | Iquitos | community | no | IgG ELISA | no | 3000 | 486 | 16.2% | rural | native |
| [65] | 2015 | Peru | Loreto | Datem del Marañon | hospital | no | IgG ELISA | DENV, ILHV, SLEV, WNV, YFV, VEEV, UNAV, EEEV, Allpahuayo virus, Tacaribe virus, bunyavirus, CARV, MAGV, MURV, OROV | 364 | 6 | 1.6% | mixed | native |
| [66] | 2017 | Peru | Amazonas |  | community | yes | Culture, IA, RT-PCR | DENV, ZIKV, VEEV, CHIKV, OROV | 1983 | 11 | 0.6% | rural | native |
| [67] | 1961-1962 | Surinam | Marowijne | Albina | community | some | HI | EEEV, DENV, SLEV, ILHV, Cache Valley virus, WEEV, YFV | 340 | 18 | 5.3% | rural | foreign |
| [53] | 1962-1964 | Surinam |  |  | community | some | HI seroconversion | VEEV, UNAV, SLEV, CARV, MUCV, Oriboca virus, Restan virus, Cache Valley virus, Paramaribo virus | 500 | 8 | 1.6% | rural | foreign/  military |

These studies were not classified in other categories but strongly indicate presence of MAYV.

*HI: hemagglutination inhibition, NT: neutralization test, SLF: Semliki Forest virus, IA: immunofluorescence assay.

**AURV: Aura virus, BUNV: Bunyawera virus, BUSV: Bussuquara virus, CARV: Caraparu virus, CHIKV: chikungunya virus, DENV: dengue virus, EEEV: Eastern equine encephalitis virus, GUAV: Guaroa virus, ILHV: Ilheus virus, MADV: Madariaga virus, MAGV: Maguari virus, MUCV: Mucambo virus, OROV: Oropuche virus, ROCV: Rocio virus, SLEV: Saint Louis encephalitis virus, UNAV: Una virus, VEEV: Venezuelan equine encephalitis virus, WEEV: Western equine encephalitis virus, YFV: yellow fever virus, ZIKV: Zika virus.

**Table I. Studies that detected MAYV in animals.**

| Family | Genus | Species | Year min | Year max | Country | State | Town/ city | Native or migratory birds | Diagnostic method* | Total | No. positive | Antibody titres | Zone | Ref |
| --- | --- | --- | --- | --- | --- | --- | --- | --- | --- | --- | --- | --- | --- | --- |
| Order primates | | | | | | | | | | | | | | |
| Aotidae | *Aotus* |  | 1957 | 1957 | Colombia | Santander | San Vicente de Chucuri |  | HI | 2 | 1 |  | rural | [48] |
| Atelidae | *Alouatta* |  | 1957 | 1957 | Colombia | Llanos Orientales |  |  | HI | 6 | 4 |  | rural | [48] |
| Atelidae | *Alouatta* |  | 1957 | 1957 | Colombia | Santander | San Vicente de Chucuri |  | HI | 5 | 3 |  | rural | [48] |
| Cebidae | *Sapajus* |  | 1957 | 1957 | Colombia | Santander | San Vicente de Chucuri |  | HI | 5 | 1 |  | rural | [48] |
| Icteridae | *Icterus* | *Icterus spurius* | 1967 | 1967 | US | Louisiana |  | migratory | HI, PRNT, viral culture by inoculation into suckling mice | 223 | 1 | yes | rural | [68] |
| Atelidae | *Alouatta* | *Alouatta villosa* | 1974 | 1976 | Panama | Panamá and Darién Provinces | Serranía de Majé |  | PRNT | 5 | 3 |  | sylvatic | [69] |
| Cebidae | *Sapajus* |  | 1978 | 1978 | Brazil | Para | Belterra |  | HI, PNRT | 1 | 1 |  | rural | [70] |
| Callitrichidae |  |  | 1978 | 1978 | Brazil | Para | Belterra |  | HI, PRNT | 119 | 32 |  | rural | [70] |
| Atelidae | *Alouatta* | *Alouatta seniculus* | 1994 | 1995 | French Guiana | Cayenne | Sinnamary |  | HI | 106 | 70 |  | sylvatic | [19] |
| Callitrichidae | *Saguinus* | *Saguinus midas* | 1994 | 1995 | French Guiana | Cayenne | Sinnamary |  | HI | 44 | 8 |  | sylvatic | [19] |
| Atelidae | *Alouatta* | *Alouatta seniculus* | 1994 | 1995 | French Guiana | Cayenne | Sinnamary |  | HI | 98 | 63 |  | sylvatic | [71] |
| Callitrichidae | *Saguinus* | *Saguinus midas* | 1994 | 1995 | French Guiana | Cayenne | Sinnamary |  | HI | 43 | 8 |  | sylvatic | [71] |
| Atelidae | *Alouatta* | *Alouatta seniculus* | 1994 | 1995 | French Guiana | Cayenne | Sinnamary |  | HI, PRNT | 98 | 51 |  | sylvatic | [72] |
| Pitheciidae | *Pithecia* | *Pithecia pithecia* | 1994 | 1995 | French Guiana | Cayenne | Sinnamary |  | HI, PRNT | 5 | 5 |  | sylvatic | [72] |
| Pitheciidae | *Pithecia* | *Pithecia pithecia* | 1994 | 1995 | French Guiana | Cayenne | Sinnamary |  | HI, PRNT | 5 | 4 |  | sylvatic | [72] |
| Cebidae | *Saimiri* | *Saimiri sciureus* | 1994 | 1995 | French Guiana | Cayenne | Sinnamary |  | HI, PRNT | 6 | 4 |  | sylvatic | [72] |
| Callitrichidae | *Saguinus* | *Saguinus midas* | 1994 | 1995 | French Guiana | Cayenne | Sinnamary |  | HI, PRNT | 42 | 8 |  | sylvatic | [72] |
| Atelidae | *Lagothrix* | *Lagothrix poeppigii* | 2007 | 2008 | Peru | Loreto | Maynas |  | ELISA, PRNT | 11 | 6 |  | rural | [49] |
| Atelidae | *Alouatta* | *Alouatta seniculus* | 2007 | 2008 | Peru | Loreto | Maynas |  | ELISA, PRNT | 1 | 1 |  | rural | [49] |
| Pitheciidae | *Cacajao* | *Cacajao calvus* | 2007 | 2008 | Peru | Loreto | Maynas |  | ELISA, PRNT | 3 | 1 |  | rural | [49] |
| Cebidae | *Sapajus* | *Sapajus macrocephalus* | 2007 | 2008 | Peru | Loreto | Maynas |  | ELISA, PRNT | 6 | 1 |  | rural | [49] |
| Cebidae | *Sapajus* | *Sapajus libidinosus* | 2008 | 2010 | Brazil | Alagoas | Maceió |  | HI | 5 | 1 | yes | captivity | [73] |
| Cebidae | *Sapajus* | *Sapajus libidinosus* | 2008 | 2010 | Brazil | Paraíba | Cabedelo |  | HI | 37 | 12 | yes | captivity | [73] |
| Cebidae | *Sapajus* | *Sapajus libidinosus* | 2008 | 2010 | Brazil | Pernambuco | Recife |  | HI | 16 | 4 | yes | captivity | [73] |
| Cebidae | *Sapajus* | *Sapajus libidinosus* | 2008 | 2010 | Brazil | Rio Grande do Norte | Natal |  | HI | 16 | 7 | yes | captivity | [73] |
| Cebidae | *Sapajus* | *Sapajus libidinosus* | 2008 | 2010 | Brazil | Piauí | Teresina |  | HI | 26 | 5 | yes | captivity | [73] |
| Cebidae | *Sapajus* | *Cebus apella* | 2009 | 2010 | Brazil | Mato Grosso do Sul | Bonito |  | HI | 35 | 7 | yes | sylvatic | [74] |
| Atelidae | *Alouatta* | *Alouatta caraya* | 2010 | 2010 | Brazil | Mato Grosso do Sul | Campo Grande |  | HI | 2 | 2 | yes | sylvatic | [74] |
| Atelidae | *Ateles* | *Ateles marginatus* | 2012 | 2017 | Brazil | Bahia | Salvador |  | HI, PRNT | 3 | 1 | yes | captivity | [75] |
| Cebidae | *Sapajus* | *Sapajus xanthosternos* | 2012 | 2017 | Brazil | Bahia | Salvador |  | HI, PRNT | 11 | 1 | yes | captivity | [75] |
| Cebidae | *Sapajus spp* |  | 2013 | 2013 | Brazil | Mato Grosso do Sul | Jardim |  | HI | 13 | 1 | yes | sylvatic | [76] |
| Order Rodentia | | | | | | | | | | | | | | |
| Dasyproctidae | *Dasyprocta* | *Dasyprocta punctata* | 1974 | 1976 | Panama | Provincia de Panamá y Provincia de Darién | Serranía de Majé |  | PRNT | 5 | 3 |  | sylvatic | [69] |
| Dasyproctidae | *Dasyprocta* | *Dasyprocta leporina* | 1994 | 1995 | French Guiana | Cayenne | Sinnamary |  | HI, PRNT | 29 | 5 |  | sylvatic | [72] |
| Erethizontidae | *Coendou* | *Coendou prehensilis* | 1994 | 1995 | French Guiana | Cayenne | Sinnamary |  | HI, PRNT | 26 | 3 |  | sylvatic | [72] |
| Erethizontidae | *Coendou* | *Coendou melanurus* | 1994 | 1995 | French Guiana | Cayenne | Sinnamary |  | HI, PRNT | 15 | 2 |  | sylvatic | [72] |
| Echimyidae | *Proechimys* |  | 1994 | 1995 | French Guiana | Cayenne | Sinnamary |  | HI, PRNT | 18 | 1 |  | sylvatic | [72] |
| Echimyidae | *Echimys* |  | 1994 | 1995 | French Guiana | Cayenne | Sinnamary |  | HI, PRNT | 21 | 1 |  | sylvatic | [72] |
| Dasyproctidae | *Dasyprocta* | *Dasyprocta fuliginosa* | 2007 | 2008 | Peru | Loreto | Maynas |  | ELISA, PRNT | 27 | 3 |  | rural | [49] |
| Cuniculidae | *Cuniculus* | *Cuniculus paca* | 2007 | 2008 | Peru | Loreto | Maynas |  | ELISA, PRNT | 10 | 1 |  | rural | [49] |
| Order Columbiformes | | | | | | | | | | | | | | |
| Columbidae |  |  | 1978 | 1978 | Brazil | Para | Belterra | native | HI, PRNT | 34 | 1 |  | rural | [70] |
| Order Caprimulgiformes | | | | | | | | | | | | | | |
| Caprimulgidae |  |  | 1978 | 1978 | Brazil | Para | Belterra | native | HI, PRNT | 5 | 1 |  | rural | [70] |
| Order Passeriformes | | | | | | | | | | | | | | |
| Dendrocolaptiae |  |  | 1978 | 1978 | Brazil | Para | Belterrra | native | HI, PRNT | 97 | 1 |  | rural | [70] |
| Formicariidae |  |  | 1978 | 1978 | Brazil | Para | Belterrra | native | HI, PRNT | 444 | 5 |  | rural | [70] |
| Pipridae |  |  | 1978 | 1978 | Brazil | Para | Belterrra | native | HI, PRNT | 229 | 1 |  | rural | [70] |
| Tyrannidae |  |  | 1978 | 1978 | Brazil | Para | Belterrra | migratory | HI, PRNT | 102 | 1 |  | rural | [70] |
| Fringillidae |  |  | 1978 | 1978 | Brazil | Para | Belterrra |  | HI, PRNT | 131 | 6 |  | rural | [70] |
| Order Pilosa | | | | | | | | | | | | | | |
| Megalonychidae | *Choloepus* | *Choloepus didactylus* | 1994 | 1995 | French Guiana | Cayenne | Sinnamary |  | HI, PRNT | 26 | 7 |  | sylvatic | [72] |
| Bradypodidae | *Bradypus* | *Bradypus tridactylus* | 1994 | 1995 | French Guiana | Cayenne | Sinnamary |  | HI, PRNT | 29 | 1 |  | sylvatic | [72] |
| Myrmecophagidae | *Tamandua* | *Tamandua tetradactyla* | 1994 | 1995 | French Guiana | Cayenne | Sinnamary |  | HI, PRNT | 26 | 6 |  | sylvatic | [72] |
| Order Cingulata | | | | | | | | | | | | | | |
| Dasypodidae | *Dasypus* | *Dasypus novemcinctus* | 1994 | 1995 | French Guiana | Cayenne | Sinnamary |  | HI, PRNT | 40 | 4 |  | sylvatic | [72] |
| Didelphidae | *Didelphis* | *Didelphis marsupialis* | 1994 | 1995 | French Guiana | Cayenne | Sinnamary |  | HI, PRNT | 29 | 1 |  | sylvatic | [72] |
| Dasypodidae | *Dasypus* | *Dasypus novemcinctus* | 2007 | 2008 | Peru | Loreto | Maynas |  | ELISA, PRNT | 4 | 2 |  | rural | [49] |
| Order Didelphimorphia | | | | | | | | | | | | | | |
| Didelphidae | *Didelphis* | *Didelphis albiventris* | 1994 | 1995 | French Guiana | Cayenne | Sinnamary |  | HI, PRNT | 19 | 2 |  | sylvatic | [72] |
| Didelphidae | *Philander* | *Philander opossum* | 1994 | 1995 | French Guiana | Cayenne | Sinnamary |  | HI, PRNT | 27 | 5 |  | sylvatic | [72] |
| Didelphidae | *Caluromys* | *Caluromys philander* | 1994 | 1995 | French Guiana | Cayenne | Sinnamary |  | HI, PRNT | 5 | 1 |  | sylvatic | [72] |
| Order Carnivora | | | | | | | | | | | | | | |
| Procyonidae | *Potos* | *Potos flavus* | 1994 | 1995 | French Guiana | Cayenne | Sinnamary |  | HI, PRNT | 9 | 1 |  | sylvatic | [72] |
| Mustelidae | *Eira* | *Eira barbara* | 1994 | 1995 | French Guiana | Cayenne | Sinnamary |  | HI, PRNT | 7 | 1 |  | sylvatic | [72] |
| Order Artiodactyla | | | | | | | | | | | | | | |
| Tayassuidae | *Pecari* | *Pecari tajacu* | 2007 | 2008 | Peru | Loreto | Maynas |  | ELISA, PRNT | 6 | 1 |  | rural | [49] |
| Order Perissodactyla | | | | | | | | | | | | | | |
| Equidae |  |  | 2009 | 2010 | Brazil | Mato Grosso do Sul | Pantanal |  | HI, PRNT | 748 | 44 | yes | rural | [77] |
| Order Crocodilia | | | | | | | | | | | | | | |
| Alligatoridae |  |  | 2009 | 2010 | Brazil | Mato Grosso do Sul | Pantanal |  | HI, PRNT | 87 | 2 | yes | sylvatic | [77] |

*****HI: hemagglutination inhibition, PRNT: plaque reduction neutralization test, ELISA: enzyme-linked immunosorbent assay.

**Supplementary results from phylogenetic analysis**

**Sequence selection and alignment**

We screened GenBank on November 15, 2019 for all published sequences of MAYV. We selected only complete or near complete genomes (> 11000 nt). Sixty-nine sequences were identified but four duplicates were excluded (TRVL 15537 KP842810 – FPY0046 KP842813 – BeAr20290 KT754168 – FPI0179 KP842816). The geographic distribution of these sequences included Bolivia (n= 6), Brazil (n = 29), French Guiana (n=2), Haiti (n= 5), Peru (n = 17) Trinidad and Tobago (n = 2), and Venezuela (n = 7). Location, source, and date of sample were retrieved from GenBank or original publications. Complete genomes were sampled between 1954 and 2015 (Table J). Sequences were aligned using the MUSCLE algorithm [78] with MEGA7 software [79] and manually edited to maintain codon homology.

**Phylogenetic signal and maximum-likelihood phylogeny inference**

We evaluated the substitution saturation by Xia test in DAMBE7 [80]. Full-genome analysis has a low saturation, meaning the sequences are sufficient to identify phylogenetic signal (Num OTU 32, Iss 0.064 vs Iss.cAsym 0.572, p < 0.001). The best-fitting nucleotide substitution model was selected with jModelTest 2 software [81,82] according to Bayesian information criterion (BIC). The maximum likelihood tree was constructed by generalized time-reversible + invariable sites + gamma 4 model (Table K) with IQ-TREE software**.** The statistical robustness of the tree topology was calculated with Ultrafast bootstrap support and the Shimodaira-Hasegawa-like approximate ratio test (SH-aLRT) with 2000 replicates was used to assess the statistical robustness of topologies for internal branching [83,84]. Strong statistical support along the branches was defined: BS > 75 and/or SH-aLRT > 95.

**Table J.** **Full genomes of MAYV included in the phylogenetic analysis.**

| GenBank number accession | Internal ID | Strain | Length bp | Country | State | Town / city | Year of collection | Source |
| --- | --- | --- | --- | --- | --- | --- | --- | --- |
| MK573246 | BOL_Uruma_1955 | Uruma | 11206 | Bolivia | Santa Cruz | Santa Cruz | 1955 | human |
| MK573245 | BOL_FSB0311_2002 | FSB0311 | 11206 | Bolivia |  |  | 2002 | human |
| KP842817 | BOL_FVB0069_2006 | FVB0069 | 11093 | Bolivia |  |  | 2006 | human |
| KP842814 | BOL_FVB0112_2006 | FVB0112 | 11099 | Bolivia |  |  | 2006 | human |
| KP842806 | BOL_FSB1131_2006 | FSB1131 | 11099 | Bolivia |  |  | 2006 | human |
| KP842805 | BOL_FSB0319_2002 | FSB0319 | 11099 | Bolivia |  |  | 2002 | human |
| MK573244 | BRA_BeH343155_1978 | BeH343155 | 11206 | Brazil |  |  | 1978 | human |
| MK573241 | BRA_BeH506151_1991 | BeH506151 | 11206 | Brazil |  |  | 1991 | human |
| MK573239 | BRA_BeH428890_1984 | BeH428890 | 11224 | Brazil |  |  | 1984 | human |
| MK573238 | BRA_BeH407_1955 | BeH407 | 11224 | Brazil |  |  | 1955 | human |
| KY618140 | BRA_BeH792430_2012 | BeH792430 | 11480 | Brazil | Para | Barcarena | 2012 | human |
| KY618139 | BRA_BeH758762_2012 | BeH758762 | 11365 | Brazil | Para | Parauapebas | 2009 | human |
| KY618138 | BRA_BeH744173_2008 | BeH744173 | 11381 | Brazil | Para | Santa Barbara do Para | 2008 | human |
| KY618137 | BRA_BeH744141_2008 | BeH744141 | 11381 | Brazil | Para | Belem | 2008 | human |
| KY618136 | BRA_BeH743921_1991 | BeH743921 | 11381 | Brazil | Para | Santa Barbara do Para | 2008 | human |
| KY618135 | BRA_BeH505465_1991 | BeH505465 | 11612 | Brazil | Para | Belem | 1991 | human |
| KY618134 | BRA_BeH504639_1991 | BeH504639 | 11512 | Brazil | Goias | Goiania | 1991 | human |
| KY618133 | BRA_BeH473130_1988 | BeH473130 | 11535 | Brazil | Para | Santarem | 1988 | human |
| KY618132 | BRA_BeH394885_1981 | BeH394885 | 11416 | Brazil | Para | Barro Branco | 1981 | human |
| KY618131 | BRA_BeH342916_1978 | BeH342916 | 11423 | Brazil | Para | Santarem | 1978 | human |
| KY618130 | BRA_BeAr757954_2011 | BeAr757954 | 11550 | Brazil | Rio Grande do Sul |  | 2011 | *Culex* sp. |
| KY618129 | BRA_BeAr505578_1991 | BeAr505578 | 11541 | Brazil | Para | Benevides | 1991 | *Haemagogus janthinomys* |
| KY618128 | BRA_BeAr344910_1978 | BeAr344910 | 11407 | Brazil | Para | Santarem | 1978 | *Haemagogus janthinomys* |
| KY618127 | BRA_BeAr20290_1960 | BeAr20290 | 11472 | Brazil |  |  | 1960 | *Haemagogus* sp. |
| KT818520 | BRA_LPV01_2014 | BR/SJRP/LPV01/2015 | 11438 | Brazil |  |  | 2014 | human |
| KP842820 | BRA_BeAr30853_1961 | BeAr30853 | 11105 | Brazil | Para |  | 1961 | *Ixodes* sp*.* |
| KP842819 | BRA_BeH256_1955 | BeH256 | 11105 | Brazil |  |  | 1955 | human |
| KP842818 | BRA_BeAr505411_1991 | BeAr505411 | 11141 | Brazil |  |  | 1991 | *Haemagogus janthinomys* |
| KP842809 | BRA_BeH186258_1970 | BeH186258 | 11099 | Brazil |  |  | 1970 | human |
| KP842804 | BRA_BeAn337622_1978 | BeAn337622 | 11099 | Brazil |  |  | 1978 | human |
| KP842803 | BRA_BeH343148_1978 | BeH343148 | 11099 | Brazil |  |  | 1978 | human |
| KP842802 | BRA_BeAn343102_1978 | BeAn343102 | 11099 | Brazil |  |  | 1978 | monkey |
| KM400591 | BRA_Acre27_2004 | Acre27 | 11273 | Brazil | Acre | Acrelandia | 2004 | human |
| MH513597 | BRA_H307_2015 | BR/Sinop/H307/2015 | 11147 | Brazil | Matto Grosso | Sinop | 2015 | human |
| KJ013266 | FRG_BNI-1_2013 | BNI-1 | 11376 | French Guiana |  |  | 2013 | human |
| DQ001069 | FRG_MAYLC | MAYLC | 11429 | French Guiana |  |  | 1996 | human |
| MK837007 | HAI_0737_2014 | Homo sapiens/Haiti-0737/2014 | 11429 | Haiti | Oest | Port-au-Prince | 2014 | human |
| MK837006 | HAI_0380_2014 | Homo sapiens/Haiti-0380/2014 | 11429 | Haiti | Oest | Port-au-Prince | 2014 | human |
| MN138459 | HAI_0729_2014 | Homo sapiens/Haiti-0729/2014 | 11429 | Haiti | Oest | Port-au-Prince | 2014 | human |
| KY985361 | HAI_1_2014 | Homo sapiens/Haiti-1/2014 | 11462 | Haiti |  |  | 2014 | human |
| KX496990 | HAI_1_2015 | Homo sapiens/Haiti-1/2015 | 11462 | Haiti |  |  | 2015 | human |
| MK573243 | PER_IQU2950_2000 | IQU2950 | 11206 | Peru | Loreto | Iquitos | 2000 | human |
| MK573242 | PER_OBS2209_1955 | OBS2209 | 11206 | Peru |  |  | 1995 | human |
| MK070491 | PER_IQT4235_1997 | IQT4235 | 11413 | Peru |  |  | 1997 | human |
| KY026200 | PER_FPI179_2011 | FPI0179 | 11093 | Peru |  |  | 2011 | human |
| KY026199 | PER_FPY0122_2011 | FPY_0122 | 11443 | Peru |  |  | 2011 | human |
| KY026198 | PER_FPY0046_2011 | FPY0046 | 11099 | Peru |  |  | 2011 | human |
| KY026197 | PER_FPI1738_2011 | FPI_1738 | 11438 | Peru |  |  | 2011 | human |
| KY026195 | PER_FPI1766_2011 | FPI_1766 | 11456 | Peru |  |  | 2011 | human |
| KP842816 | PER_FPI0179_2011 | FPI0179 | 11093 | Peru |  |  | 2011 | human |
| KP842815 | PER_FPI1761_2011 | FPI1761 | 11093 | Peru |  |  | 2011 | human |
| KP842813 | PER_FPY0046_2011 | FPY0046 | 11099 | Peru |  |  | 2011 | human |
| KP842812 | PER_FMD3213_2010 | FMD3213 | 11099 | Peru |  |  | 2010 | human |
| KP842811 | PER_FMD0641_2005 | FMD0641 | 11099 | Peru |  |  | 2005 | human |
| KP842808 | PER_IQU3056_2000 | IQU3056 | 11099 | Peru |  |  | 2000 | human |
| KP842807 | PER_Ohio_1995 | Ohio | 11099 | Peru |  |  | 1995 | human |
| KP842801 | PER_IQE2777_2006 | IQE2777 | 11099 | Peru |  |  | 2006 | human |
| KP842800 | PER_ARV0565_1995 | ARV0565 | 11099 | Peru |  |  | 1995 | human |
| MK573240 | TRI_TRVL15537_1957 | TRVL15537 | 11202 | Trinidad and Tobago |  |  | 1957 | *Manzonia venezuelensis* |
| MK070492 | TRI_TRVL 4675_1954 | TRVL 4675 | 11424 | Trinidad and Tobago |  |  | 1954 | human |
| MK288026 | VEN_1_2016 | Homo sapiens/Venezuela-1/2016 | 11441 | Venezuela | Portuguesa | Ospino | 2016 | human |
| KP842799 | VEN_MAYV15A_2010 | MAYV15A | 11099 | Venezuela | Portuguesa | Ospino | 2010 | human |
| KP842798 | VEN_MAYV14A_2010 | MAYV14A | 11099 | Venezuela | Portuguesa | Ospino | 2010 | human |
| KP842797 | VEN_MAYV13A_2010 | MAYV13A | 11099 | Venezuela | Portuguesa | Ospino | 2010 | human |
| KP842796 | VEN_MAYV12A_2010 | MAYV12A | 11099 | Venezuela | Portuguesa | Ospino | 2010 | human |
| KP842795 | VEN_MAYV11A_2010 | MAYV11A | 11099 | Venezuela | Portuguesa | Ospino | 2010 | human |
| KP842794 | VEN_MAYV16A_2010 | MAYV16A | 11099 | Venezuela | Portuguesa | Ospino | 2010 | human |

**Table K. Nucleotide substitution models.** The best-fitting model is in bold.

| Model | Number of estimated parameters | Log likelihood | BIC |
| --- | --- | --- | --- |
| GTR+I+G | **138** | **-38644.6** | **78574** |
| GTR+G | 137 | -38652.8 | 78581 |
| GTR+I | 137 | -38668.8 | 78613 |
| SYM+I+G | 135 | -38687.9 | 78632 |
| SYM+G | 134 | -39695.6 | 78639 |
| SYM+I | 134 | -38712.3 | 78672 |
| TrN+I+G | 135 | -38717.8 | 78692 |
| TrN+G | 134 | -38725.5 | 78698 |
| TIM1+I+G | 136 | -38717.7 | 78701 |

**References**

1. Pinheiro F, Freitas R, Travassos da Rosa J, Gabbay Y, Mello W, LeDuc J. An outbreak of Mayaro virus disease in Belterra, Brazil I. Clinical and virological findings. Am J Trop Med Hyg. 1981;30:674-81.

2. Ferguson N, Cucunubá Z, Dorigatti I, Nedjati-Gilani G, Donnelly C, Basáñez M, et al. Countering Zika in Latin America. Science. 2016;353(6297):353-4.

3. Lessler J, Ott C, Carcelen A, Konikoff J, Williamson J, Bi Q, et al. Times to key events in Zika virus infection and implications for blood donation: a systematic review. Bull World Health Organ. 2016;94:841-9.

4. Reich N, Lessler J, Cummings D, Brookmeyer R. Estimating incubation period distributions with coarse data. Stat Med. 2009;28(22):2769-84.

5. Aitken T. Virus transmission studies with Trinidadian mosquitoes. Caribbean Medical Journal. 1957;6:137-40.

6. Brustolin M, Pujhari S, Henderson C, Rasgon J. Anopheles mosquitoes may drive invasion and transmission of Mayaro virus across geographically diverse regions. PLoS Negl Trop Dis. 2018;12(e0006895).

7. Long K, Ziegler S, Thangamani S, Hausser N, Kochel T, Higgs S, et al. Experimental transmission of Mayaro virus by Aedes aegypti. Am J Trop Med Hyg. 2011;85:750-7.

8. Smith G, Francy D. Laboratory studies of a Brazilian strain of Aedes albopictus as a potential vector of Mayaro and Oropouche viruses. Journal of the American Mosquito Control Association. 1991;7:89-93.

9. Wiggins K, Eastmond B, Alto B. Transmission potential of Mayaro virus in Florida Aedes aegypti and Aedes albopictus mosquitoes. Medical and Veterinary Entomology. 2018;32:436-42.

10. Cori A, Cauchemez S, Ferguson N, Fraser C, Dahlqwist E, Alex Demarsh P, et al. EpiEstim: Estimate Time Varying Reproduction Numbers from Epidemic Curves. CRAN. 2.2-3 ed2020.

11. Cori A, Ferguson N, Fraser C, Cauchemez S. A New Framework and Software to Estimate Time-Varying Reproduction Numbers During Epidemics. American Journal of Epidemiology. 2013;178(9):1505-12.

12. Theilacker C, Held J, Allering L, Emmerich P, Schmidt-Chanasit J, Kern W, et al. Prolonged polyarthralgia in a German traveller with Mayaro virus infection without inflammatory correlates. BMC Infectious Diseases. 2013;13:369.

13. Coimbra T, Santos C, Suzuki A, Petrella S, Bisordi I, Nagamori A, et al. Mayaro virus: imported cases of human infection in São Paulo State, Brazil. Rev Inst Med trop S Paulo. 2007;49(4):221-4.

14. Terzian A, Auguste A, Vedovello D, Ferreira M, da Silva-Nunes M, Sperança M, et al. Isolation and characterization of Mayaro virus from a human in Acre, Brazil. Am J Trop Med Hyg. 2015;92(2):401-4.

15. Receveur M, Grandadam M, Pistone T, Malvy D. Infection with Mayaro virus in a French traveller returning from the Amazon region, Brazil, January, 2010. Euro Surveill. 2010;15(18):pii=19563.

16. Slegers C, Keuter M, Günther S, Schmidt-Chanasit J, van der Ven A, de Mast Q. Persisting arthralgia due to Mayaro virus infection in a traveler from Brazil: is there a risk for attendants to the 2014 FIFA World Cup? J Clin Virol. 2014;60(3):317-9.

17. Mota M, Vedovello D, Estofolete C, Malossi C, Araújo J, Nogueira M. Complete Genome Sequence of Mayaro Virus Imported from the Amazon Basin to Sao Paulo State, Brazil. Genome Announc. 2015;3(6):e01341-15.

18. Estofolete C, Mota M, Vedovello D, Nunes de Góngora D, Maia I, Nogueira M. Mayaro fever in an HIV-infected patient suspected of having Chikungunya fever. Rev Soc Bras Med Trop. 2016;49(5):648-52.

19. Talarmin A, Chandler L, Kazanji M, de Thoisy B, Debon P, Lelarge J, et al. Mayaro virus fever in French Guiana: isolation, identification, and seroprevalence. Am J Trop Med Hyg. 1998;59(3):452-6.

20. Junt T, Heraud J, Lelarge J, Labeau B, Talarmin A. Determination of natural versus laboratory human infection with Mayaro virus by molecular analysis. Epidemiol Infect. 1999;123(5):511-3.

21. Bourée P, Fichet G, Lepeytre D, Bisaro F. Le virus Mayaro: une virose guyanaise émergente. Revue Francophone des Laboratoires. 2013(456):87-9.

22. Friedrich-Jänicke B, Emmerich P, Tappe D, Günther S, Cadar D, Schmidt-Chanasit J. Genome analysis of Mayaro virus imported to Germany from French Guiana. Emerg Infect Dis. 2014;20(7):1255-7.

23. Llagonne-Barets M, Icard V, Leparc-Goffart I, Prat C, Perpoint T, André P, et al. A case of Mayaro virus infection imported from French Guiana. J Clin Virol. 2016;77:66-8.

24. White S, Mavian C, Elbadry M, Beau de Rochars V, Paisie T, Telisma T, et al. Detection and phylogenetic characterization of arbovirus dual-infections among persons during a chikungunya fever outbreak, Haiti 2014. PLoS Negl Trop Dis. 2018;12(5):e0006505.

25. Navarrete-Espinosa J, Gómez-Dantés H. Arbovirus causales de fiebre hemorrágica en pacientes del Instituto Mexicano del Seguro Social. Rev Med Inst Mex Seguro Soc. 2006;44(4):347-53.

26. Tesh R, Watts D, Russell K, Damodaran C, Calampa C, Cabezas C, et al. Mayaro Virus Disease: An Emerging Mosquito‐Borne Zoonosis in Tropical South America. Clinical Infectious Diseases. 1999;28(1):67-73.

27. Neumayr A, Gabriel M, Fritz J, Günther S, Hatz C, Schmidt-Chanasit J, et al. Mayaro virus infection in traveler returning from Amazon Basin, northern Peru. Emerg Infect Dis. 2012;18(4):695-6.

28. Hassing R, Leparc-Goffart I, Blank S, Thevarayan S, Tolou H, van Doornum G, et al. Imported Mayaro virus infection in the Netherlands. J Infect. 2010;61(4):343-5.

29. Anderson C, Downs W, Wattley G, Ahin N, Reese A. Mayaro Virus: A New Human Disease Agent II. Isolation from Blood of Patients in Trinidad, B.W.I.1. Am J Trop Med Hyg. 1957;6(6):1012-6.

30. Torres J, Russell K, Vasquez C, Tesh R, Salas R, Watts D. Family cluster of Mayaro fever, Venezuela. Emerg Infect Dis. 2004;10(7):1304-6.

31. Forshey B, Guevara C, Laguna-Torres V, Cespedes M, Vargas J, Gianella A, et al. Arboviral Etiologies of Acute Febrile Illnesses in Western South America, 2000–2007. PLoS Negl Trop Dis. 2010;4(8):e787.

32. de Figueiredo R, Thatcher B, de Lima M, Almeida T, Alecrim W, de Farias Guerra M. Doenças exantemáticas e primeira epidemia de dengue ocorridaem Manaus, Amazonas, no período de 1998-1999. Revista da Sociedade Brasileira de Medicina 2004;37(6):476-9.

33. Mourão M, de Souza Bastos M, de Figueiredo R, Gimaque J, dos Santos Galusso E, Kramer V, et al. Mayaro Fever in the City of Manaus, Brazil, 2007–2008. Vector Borne Zoonotic Dis. 2012;12(1):42-6.

34. Nunes M, Barbosa T, Casseb L, Neto J, Segura N, Monteiro H, et al. Eco-epidemiologia dos arbovírus na área de influência da rodovia Cuiabá-Santarém (BR 163), Estado do Pará, Brasil. Cad Saúde Pública. 2009;25(12):2583-602.

35. da Costa V, de Rezende Féres V, Saivish M, de Lima Gimaque B, Moreli M. Silent emergence of Mayaro and Oropouche viruses in humans in Central Brazil. Int J Infect Dis. 2017;62:84-5.

36. Vieira CdS, da Silva D, Barreto E, Siqueira E, Colombo T, Ozanic K, et al. Detection of Mayaro virus infections during a dengue outbreak in Mato Grosso, Brazil. Acta Trop. 2015;147:12-6.

37. Zuchi N, da Silva Heinen L, dos Santos M, Pereira F, Slhessarenko R. Molecular detection of Mayaro virus during a dengue outbreak in the state of Mato Grosso, Central-West Brazil. Mem Inst Oswaldo Cruz. 2014;109(6):820-3.

38. Brunini S, França D, Silva J, Silva L, Silva F, Spadoni M, et al. High Frequency of Mayaro Virus IgM among Febrile Patients, Central Brazil. Emerg Infect Dis. 2017;23(6):1025-6.

39. de Souza Costa M, Siqueira Maia L, Costa de Souza V, Gonzaga A, Correa de Azevedo V, Ramos Martins L, et al. Arbovirus investigation in patients from Mato Grosso during Zika and Chikungunya virus introdution in Brazil, 2015–2016. Acta Trop. 2019;190:395-402.

40. Teixeira Barros E. Caracterização molecular de chikungunya virus e investigação dos arbovírus dengue virus e Mayaro virus no estado do Piauí. Parnaíba, Brazil: Universidade Federal do Piauí; 2018.

41. Mutricy R, Epelboin L, Mosnier E, Matheus S, Djossou F, Rousset D. TROP-11 - Caractéristiques clinico-biologiques d’une arbovirose méconnue en Guyane française : le virus mayaro. Médecine et maladies infectieuse. 2016;46(4):106.

42. Instituto Nacional de Salud Perú. Perfil etiológico del síndrome febril en áreas de alto riesgo de transmisión de enfermedades infecciosas de impacto en salud pública en el Perú, 2000-2001. Rev perú med exp salud publica. 2005;22(3):165-74.

43. Halsey E, Siles C, Guevara C, Vilcarromero S, Jhonston E, Ramal C, et al. Mayaro virus infection, Amazon Basin region, Peru, 2010-2013. Emerg Infect Dis. 2013;19(11):1839-42.

44. Izurieta R, Macaluso M, Watts D, Tesh R, Guerra B, Cruz L, et al. Hunting in the rainforest and Mayaro virus infection: An emerging alphavirus in Ecuador. J Glob Infect Dis. 2011;3(4):317-23.

45. Abad-Franch F, Grimmer G, de Paula V, Figueiredo L, Braga W, Luz S. Mayaro virus infection in Amazonia: a multimodel inference approach to risk factor assessment. PLoS Negl Trop Dis. 2012;6(10):e1846.

46. Black F, Hierholzer W, Pinheiro F, Evans A, Woodall J, Opton E, et al. Evidence for persistence of infectious agents in isolated human populations. American Journal of Epidemiology. 1974;100(3):230-50.

47. Downs W, Anderson C. Distribution of Immunity to Mayaro Virus Infection in West Indies. West Indian Medical Journal. 1958;7(3):190-4.

48. Groot H. Estudios sobre virus transmitidos por artrópodos en Colombia. Rev Acad Colomb Cienc Ex Fis Nat. 1964;12:191-217.

49. Pérez J, Carrera J, Serrano E, Pittí Y, Maguiña J, Mentaberre G, et al. Serologic Evidence of Zoonotic Alphaviruses in Humans from an Indigenous Community in the Peruvian Amazon. Am J Trop Med Hyg. 2019;101(6):1212-8.

50. Prías-Landínez E, Bernal-Cúbides C, de Torres S, Romero-León M. Encuesta serológica de virus transmitidos por artrópodos. Bogotá, Colombia: Instituto Nacional de Salud; 1970.

51. Spence L, Downs W. Virological investigations in Guyana, 1956-1966. West Indian Medical Journal. 1968;17:83-9.

52. van Tongeren H. Arbovirus group A spectrum in the province of Brokopondo, Surinam. A serological survey. Trop Geogr Med. 1965;17:172-85.

53. Jonkers A, Spence L, Karbaat J. Arbovirus infections in Dutch military personnel stationed in Surinam: Further studies. Trop Geogr Med. 1968;20:251-6.

54. Buckley S, Davis III J, Madalengoitia J, Flores W, Casals J. Arbovirus neutralization tests with Peruvian sera in Vero cell cultures. Bull World Health Organ. 1972;46(4):451-5.

55. Taylor S, Patel P, Herold T. Recurrent arthralgias in a patient with previous Mayaro fever infection. South Med J. 2005;98(4):484-5.

56. Causey O, Maroja O. Mayaro virus: a new human disease agent. III. Investigation of an epidemic of acute febrile illness on the river Guama in Pará, Brazil, and isolation of Mayaro virus as causative agent. Am J Trop Med Hyg. 1957;6(6):1017-23.

57. Neel J, Andrade A, Brown G, Eveland W, Goobar J, Sodeman Jr W, et al. Further Studies of the Xavante Indians. IX. Immunologic Status with Respect to Various Diseases and Organisms. Am J Trop Med Hyg. 1968;17(3):486-98.

58. Pinheiro F, Bensabath G, Rosa A, Lainson R, Shaw J, Ward R, et al. Public Health Hazards Among Workers Along The Trans-Amazon Highway. Journal of Occupational Medicine. 1977;19(7):490-7.

59. Tavares-Neto J, da Rosa A, Vasconcelos P, Costa J, da Rosa J, Marsden P. Pesquisa de anticorpos para arbovírus no soro de residentes no povoado de Corte de Pedra, Valença, Bahia. Mem Inst Oswaldo Cruz. 1986;81(4):351-8.

60. Tavares-Neto J, Freitas-Carvalho J, Nunes M, Rocha G, Rodrigues S, Damasceno E, et al. Pesquisa de anticorpos contra arbovírus e o vírus vacinal de febre amarela em uma amostra da população de Rio Branco, antes e três meses após a vacina 17D. Revista da Sociedade Brasileira de Medicina Tropical. 2004;37:1-6.

61. Cruz A, dos Prazeres A, Gama E, de Lima M, Azevedo R, Casseb L, et al. Vigilância sorológica para arbovírus em Juruti, Pará, Brasil. Cad Saúde Pública. 2009;25(11):2517-23.

62. Carrera J, Bagamian K, Travassos da Rosa A, Wang E, Beltran D, Gundaker N, et al. Human and Equine Infection with Alphaviruses and Flaviviruses in Panama during 2010: A Cross-Sectional Study of Household Contacts during an Encephalitis Outbreak. Am J Trop Med Hyg. 2018;98(6):1798-804.

63. Madalengoitia J, Flores W, Casals J. Arbovirus antibody survey of sera from residents of Eastern Peru. Pan American Health Organization; 1973. Contract No.: 4.

64. Long K, Morrison A, Forshey B, Huaman A, Rocha C, Carrion R, et al. Seroprevalence rates of Mayaro virus in urban and rural areas of Maynas Province, Peru. Am J Trop Med Hyg. 2008;79(6 Suppl):92.

65. Guevara C, Jhonston E, Huaman A, Silva M, Gómez J, Laguna-Torres V, et al. First evidence of Una virus infections in indigenous and non-indigenous communities in Loreto, Peru. Am J Trop Med Hyg. 2015;93(4 Suppl):240.

66. Coaguila M, Garcia M, Figueroa M, Merino N, Marcelo A, Cobos M, et al. Re-emerging of Mayaro virus in areas with circulation of dengue virus in the Peruvian Amazon. Am J Trop Med Hyg. 2017;97(5 Suppl):2-3.

67. Karbaat J, Jonkers A, Spence L. Arbovirus infections in Dutch military personnel stationed in Surinam. A preliminary study. Trop Geogr Med. 1964;16:370-6.

68. Calisher C, Gutiérrez E, Maness K, Lord R. Isolation of Mayaro virus from a migrating bird captured in Louisiana in 1967. Bull Pan Am Health Organ. 1974;8(3):243-8.

69. Seymour C, Peralta P, Montgomery G. Serologic evidence of natural togavirus infections in Panamanian sloths and other vertebrates. Am J Trop Med Hyg. 1983;32:854-61.

70. Hoch A, Peterson N, LeDuc J, Pinheiro F. An outbreak of Mayaro virus disease in Belterra, Brazil. III. Entomological and ecological studies. Am J Trop Med Hyg. 1981;30:689-98.

71. de Thoisy B, Vogel I, Reynes J, Pouliquen J, Carme B, Kazanji M, et al. Health evaluation of translocated free-ranging primates in French Guiana. Am J Primatol. 2001;54:1-16.

72. de Thoisy B, Gardon J, Salas R, Morvan J, Kazanji M. Mayaro virus in wild mammals, French Guiana. Emerg Infect Dis. 2003;9:1326-9.

73. Laroque P, Valença-Montenegro M, Ferreira D, Chiang J, Corderio M, Vasconcelos P, et al. Levantamento soroepidemiológico para arbovírus em macaco-prego-galego (Cebus flavius) de vida livre no Estado da Paraíba e em macaco-prego (Cebus libidinosus) de cativeiro do Nordeste do Brasil. Pesq Vet Bras. 2014;34(5):462-8.

74. Batista P, Andreotti R, Chiang J, Ferreira M, da Costa Vasconcelos P. Seroepidemiological monitoring in sentinel animals and vectors as part of arbovirus surveillance in the state of Mato Grosso do Sul, Brazil. Rev Soc Bras Med Trop. 2012;45(2):168-73.

75. Moreira-Soto A, de Oliveira Carneiro I, Fischer C, Feldmann M, Kümmerer B, Silva N, et al. Limited Evidence for Infection of Urban and Peri-urban Nonhuman Primates with Zika and Chikungunya Viruses in Brazil. mSphere. 2018;3(1):e00523-17.

76. Batista P, Andreotti R, de Almeida P, Marques A, Rodrigues S, Chiang J, et al. Detection of arboviruses of public health interest in free-living New World primates (Sapajus spp.; Alouatta caraya) captured in Mato Grosso do Sul, Brazil. Rev Soc Bras Med Trop. 2013;46(6):684-90.

77. Pauvolid-Corrêa A, Juliano R, Campos Z, Velez J, Nogueira R, Komar N. Neutralising antibodies for Mayaro virus in Pantanal, Brazil. Mem Inst Oswaldo Cruz. 2015;110(1):125-33.

78. Edgar R. MUSCLE: Multiple sequence alignment with high accuracy and high throughput. Nucleic Acids Res. 2004;32(5):1792-7.

79. Kumar S, Stecher G, Tamura K. MEGA7: Molecular Evolutionary Genetics Analysis Version 7.0 for Bigger Datasets. Mol Biol Evol. 2016;33(7):1870-4.

80. Xia X. DAMBE7: New and Improved Tools for Data Analysis in Molecular Biology and Evolution. Mol Biol Evol. 2018;35(6):1550-2.

81. Darriba D, Taboada G, Doallo R, Posada D. jModelTest 2: more models, new heuristics and parallel computing. Nature Methods. 2012;9(8):772.

82. Guindon S, Gascuel O. A simple, fast, and accurate algorithm to estimate large phylogenies by maximum likelihood. Syst Biol. 2003;52(5):696-704.

83. Nguyen L, Schmidt H, von Haeseler A, Minh B. IQ-TREE: A Fast and Effective Stochastic Algorithm for Estimating Maximum-Likelihood Phylogenies. Mol Biol Evol. 2015;32(1):268-74.

84. Trifinopoulos J, Nguyen L, von Haeseler A, Minh B. W-IQ-TREE: a fast online phylogenetic tool for maximum likelihood analysis. Nucleic Acids Res. 2016;44:W232-5.
