# Supplementary material for: The epidemiology of Mayaro virus in the Americas: A systematic review and key parameter estimates for outbreak modelling
Source: PLoS Negl Trop Dis. 2021 Jun 3;15(6):e0009418. doi: 10.1371/journal.pntd.0009418 (PMC8205173; doi:10.1371/journal.pntd.0009418)
Supplement: Attachment — Submitted filename: MAYV peer review response_ZC_KC_ZC_15M.pdf [file pntd.0009418.s004.pdf]

Imperial College London

MRC Centre for Global Infectious Disease Analysis
Department of Infectious Disease Epidemiology
Imperial College London
St Mary's Campus, Norfolk Place
London W2 1PG
United Kingdom

zulma.cucunuba@imperial.ac.uk

16th March 2021

Zulma M. Cucunubá, MD PhD
Postdoctoral Fellow

Eugenia Corrales-Aguilar, PhD

Deputy Editor

PLOS Neglected Tropical Diseases

Dear Dr. Corrales-Aguilar and Reviewers,

Thank you for comments related to our manuscript entitled "**The epidemiology of Mayaro virus in the Americas: A systematic review and key parameter estimates for outbreak modelling.**" We greatly appreciate the insights of the peer reviewers and have carefully considered each comment. We believe the paper is stronger as a result and hope you find the following changes satisfactory.

Reviewer #1

Methods

1/ The choice to exclude studies not in English, French, Spanish or Portuguese requires justification. This may not be all that difficult as I'd think most if not all important studies would be available in these languages, but nonetheless scientific (as above) and/or pragmatic (language fluency among the authors) justification should be presented.

> We thank Reviewer #1 for this comment. We have now clarified that one paper in Chinese was excluded due to lack of fluency among the authors (lines 110-112).

2/ The meaning of the statement that "Each study was allocated to two reviewers who independently screened abstracts and titles" (lines 107-8), most specifically the meaning of "study" in this context. I'd infer that this means the areas covered in the paper ("(i) the time of exposure to MAYV; (ii) the time of symptom onset....") but the statement could be misconstrued as written.

> We have changed the word "study" to "article," as we included both peer-reviewed publications as well as grey literature. Indeed, one article can include more than one "study" or experiment. We apologize for any confusion (lines 108-9).

Results

1/ Figure 3 needs slight editing as some of the data points (coloured circles) have been clipped at the bottom or top.

> We thank Review #1 for pointing this out, and we have edited the figure accordingly.

2/ The text above Table 3 includes all (or perhaps nearly all) the results from the table. These results should be in the table or text, but not both (at least not in full).

We fully agree with this comment and have shortened the text above the table (lines 269-271).

3/ What are "Ileus antibodies" (Table 1). I'm not sure if I should know, but I don't. If this is not widely known an explanation should be given (or if it's a typo it should be corrected).

We thank Reviewer #1 for astutely catching this typo. We meant to write “Ilheus virus antibodies” and have corrected it. Ilheus virus is a flavivirus. It also appears in some of the tables in the supplement (line 462).

Summary and General Comments

I wonder if you could update the literature search beyond 11 January 2019?

We thank Reviewer #1 for this comment, but unfortunately do not think it would be feasible as nearly all of the authors are currently working on the COVID-19 pandemic. Since January 2019, Google Scholar alone is showing 1,210 results for Mayaro Virus or MAYV.

Reviewer #2

1. Introduction Lines 62-64. The sentence on serological cross-reactivity seems out of place here. I would recommend addressing this point around line 85 where the authors discuss the difficulty of diagnosis.

We thank Reviewer #2 for this recommendation and have moved the sentence (lines 82-85).

2. Page 16 Table 3. The parameters are assumed to be the same for all studies. I was wondering if we could expect variations of the epidemiological parameters, for instance with the climatic conditions. (Mordecai Plos NTD 2013) discuss the impact of temperature on arbovirus transmission. Can we expect the mosquito lifetime to depend on the temperature and the season? If so, how would it change the different parameters?

Yes, we expect the parameters that depend on the vector, such as the extrinsic incubation period and mosquito lifetime, to change with temperature. We have added a section to the discussion to address this comment more fully (lines 386-394).

Although the generation time distribution here was estimated independently of temperature, the component parameters that depend on the vector can vary based on temperature. For example, as temperature increases within a range acceptable to the vector, dengue virus replicates faster, decreasing the extrinsic incubation period (EIP) of Ae. aegypti [70]. Additionally, mosquito longevity depends on temperature and species [71]. It is unclear which species of mosquito is most important in MAYV transmission. Moreover, due to limited data, we had to pool information on six different species of mosquitoes to estimate the EIP. Thus, in order to estimate a

temperature-dependent generation time distribution for MAYV, more vector data is needed.

3. Figure 5 Page 17. The authors applied serocatalytic models to assess the force of infection (FOI) from age stratified seroprevalence studies. They tested models of time-independent (endemicity) and time-dependent FOI. However, other models could explain the data, and from cross-sectional seroprevalence studies it is not possible to distinguish variations in time of the FOI or different exposures with age. For instance, the curve from Ecuador 1997 could also be due to an outbreak that happened in the 1990s and where adults were more exposed than children. It would be good to discuss this possibility, even more so that MAYV is often considered, as yellow fever, to be more likely to infect adults that are active near forested areas.

We agree with this comment and thank the Reviewer for mentioning it. We address the limitation of the cross-sectional data in lines 467-473.

Another limitation is that the use of cross-sectional seroprevalence studies did not allow us to investigate changes in the FOI over time or different exposures by age. One assumption of the catalytic models applied in our analysis is that there are no differences in exposure by age, which seems plausible when assuming no previous exposure such as in Brazil 2017 (Fig 5B). However, we acknowledge that in some situations, adults may have had higher exposure to MAYV than children due to working exposure such as in forested areas. Unfortunately, we do not have access to such data.

Reviewer #3:

Minor suggestions:
Details of the protocols are provided in the main text. Even though the used model is widely used by the community, details about the applied equations and model per se could be included in the Support Information.

We appreciate this comment. Serocatalytic models have been explained in the main text (lines 124-131) and, we have added a reference to line 125. Also, equations for generation time and R calculations have been included in main text (lines 139-152) and expanded in Supplementary Information. Methods for Phylogenetic analysis have been included in lines 154-164.

The authors used several different software for performing the simulation and for building the phylogenetic tree. It would be useful for the community if the codes and scripts used in these calculations to be provided in the SI.

We thank the Reviewer for this suggestion. We have provided the relevant codes and scripts for the different analyses in the following GitHub repository: https://github.com/zmcucunuba/mayv-review.

Also, the data extracted from the literature and organized could also be made available for reproducibility.

We thank the Reviewer for this suggestion and have added the data to the following GitHub repository: https://github.com/zmcucunuba/mayv-review.

I suggest increasing the font size of all Figures' text to help the reader.

We thank the Reviewer for this comment and have increased the font size of all Figures.

The authors should expand the discussion about the possible overestimation of the Mayaro R0 due to the serological cross-reactivity with other viruses.

We appreciate this comment. We have expanded the discussion as requested in lines 457-464.

Consequently, the MAYV R_0 from seroprevalence studies may have been overestimated due to serological cross-reactivity among other circulating alphaviruses. In contrast, cross-reactivity likely did not affect the estimation of R_0 from the MAYV outbreak in Bolivia. The individuals affected by the outbreak were migrants from Japan and would have had few opportunities to be infected by other alphaviruses. Although asymptomatic individuals tested positive for yellow fever virus and Ilheus virus antibodies, these viruses are flaviviruses and therefore are not expected to affect the immune response to an alphavirus.

Finally, we fixed a few small grammatical errors in the main text and supplement.

Thank you,

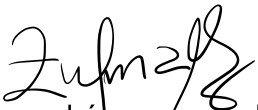

Zulma Cucunubá, corresponding author
