# Supplementary material for: The epidemiology of Mayaro virus in the Americas: A systematic review and key parameter estimates for outbreak modelling
Source: PLoS Negl Trop Dis. 2021 Jun 3;15(6):e0009418. doi: 10.1371/journal.pntd.0009418 (PMC8205173; doi:10.1371/journal.pntd.0009418)
Supplement: Attachment — Submitted filename: ReviewMayaro.docx [file pntd.0009418.s003.docx]

This paper proposes a study of the epidemiological parameters related to the transmission of MAYV using past reports of outbreaks, cases and serological studies. I greatly appreciated this paper. It is very clearly written and one of its strength is that the authors applied a variety of models to exploit the information available in the literature. This allowed them to quantify epidemiological parameters that had never been estimated before for MAYV.

I recommend publication of the manuscript after some minor revisions:

1. Introduction Lines 62-64. The sentence on serological cross-reactivity seems out of place here. I would recommend addressing this point around line 85 where the authors discuss the difficulty of diagnosis.
2. Page 16 Table 3. The parameters are assumed to be the same for all studies. I was wondering if we could expect variations of the epidemiological parameters, for instance with the climatic conditions. (Mordecai Plos NTD 2013) discuss the impact of temperature on arbovirus transmission. Can we expect the mosquito lifetime to depend on the temperature and the season? If so, how would it change the different parameters?
3. Figure 5 Page 17. The authors applied serocatalytic models to assess the force of infection (FOI) from age stratified seroprevalence studies. They tested models of time-independent (endemicity) and time-dependent FOI. However, other models could explain the data, and from cross-sectional seroprevalence studies it is not possible to distinguish variations in time of the FOI or different exposures with age. For instance, the curve from Ecuador 1997 could also be due to an outbreak that happened in the 1990s and where adults were more exposed than children. It would be good to discuss this possibility, even more so that MAYV is often considered, as yellow fever, to be more likely to infect adults that are active near forested areas.
